# Advancing diagnosis in a cardiac arrest case and suspected MINOCA: the complementary roles of imaging, biopsy, and genetic testing

**DOI:** 10.1093/ehjimp/qyae135

**Published:** 2024-12-14

**Authors:** Giuseppe Ciliberti, Paolo Compagnucci, Michela Casella, Francesco Schiavone, Monica De Gaspari, Stefania Rizzo, Cristina Basso, Andrea Giovagnoni, Federico Guerra, Giada Tortora, Antonio Dello Russo

**Affiliations:** Cardiology and Arrhythmology Clinic, Marche University Hospital, Via Conca 71, 60020 Ancona, Italy; Department of Biomedical Sciences and Public Health, Marche Polytechnic University, Via Tronto, 10/a, 60126 Ancona, Italy; Cardiology and Arrhythmology Clinic, Marche University Hospital, Via Conca 71, 60020 Ancona, Italy; Department of Biomedical Sciences and Public Health, Marche Polytechnic University, Via Tronto, 10/a, 60126 Ancona, Italy; Cardiology and Arrhythmology Clinic, Marche University Hospital, Via Conca 71, 60020 Ancona, Italy; Department of Clinical, Special and Dental Sciences, Marche Polytechnic University, 60126 Ancona, Italy; Cardiology and Arrhythmology Clinic, Marche University Hospital, Via Conca 71, 60020 Ancona, Italy; Department of Biomedical Sciences and Public Health, Marche Polytechnic University, Via Tronto, 10/a, 60126 Ancona, Italy; Cardiovascular Pathology Unit, Department of Cardiac, Thoracic, Vascular Sciences and Public Health, Azienda Ospedaliera-University of Padua, 35128 Padova, Italy; Cardiovascular Pathology Unit, Department of Cardiac, Thoracic, Vascular Sciences and Public Health, Azienda Ospedaliera-University of Padua, 35128 Padova, Italy; Cardiovascular Pathology Unit, Department of Cardiac, Thoracic, Vascular Sciences and Public Health, Azienda Ospedaliera-University of Padua, 35128 Padova, Italy; Department of Clinical, Special and Dental Sciences, Marche Polytechnic University, 60126 Ancona, Italy; Department of Radiology, University Hospital “Ospedali Riuniti”, 60126 Ancona, Italy; Cardiology and Arrhythmology Clinic, Marche University Hospital, Via Conca 71, 60020 Ancona, Italy; Department of Biomedical Sciences and Public Health, Marche Polytechnic University, Via Tronto, 10/a, 60126 Ancona, Italy; Medical Genetic Unit, Azienda Ospedaliero-Universitaria delle Marche, 60126 Ancona, Italy; Cardiology and Arrhythmology Clinic, Marche University Hospital, Via Conca 71, 60020 Ancona, Italy; Department of Biomedical Sciences and Public Health, Marche Polytechnic University, Via Tronto, 10/a, 60126 Ancona, Italy

**Keywords:** OHCA, MINOCA, endomiocardial biopsy, electroanatomical mapping, cardiac magnetic resonance, genetic

A 45-year-old man without family history of sudden cardiac death presented to the emergency department after out-of-hospital cardiac arrest which required repeated DC-shocks for termination of recurrent ventricular fibrillation. For the presence of ST-segment elevation in the inferior leads at ECG (*Panel A*), an emergency coronary angiography was scheduled, which ruled out obstructive lesions (*Panels B* and *C*) and signs of plaque disruption on intravascular ultrasound (*Panels D* and *E*).

**Figure:**
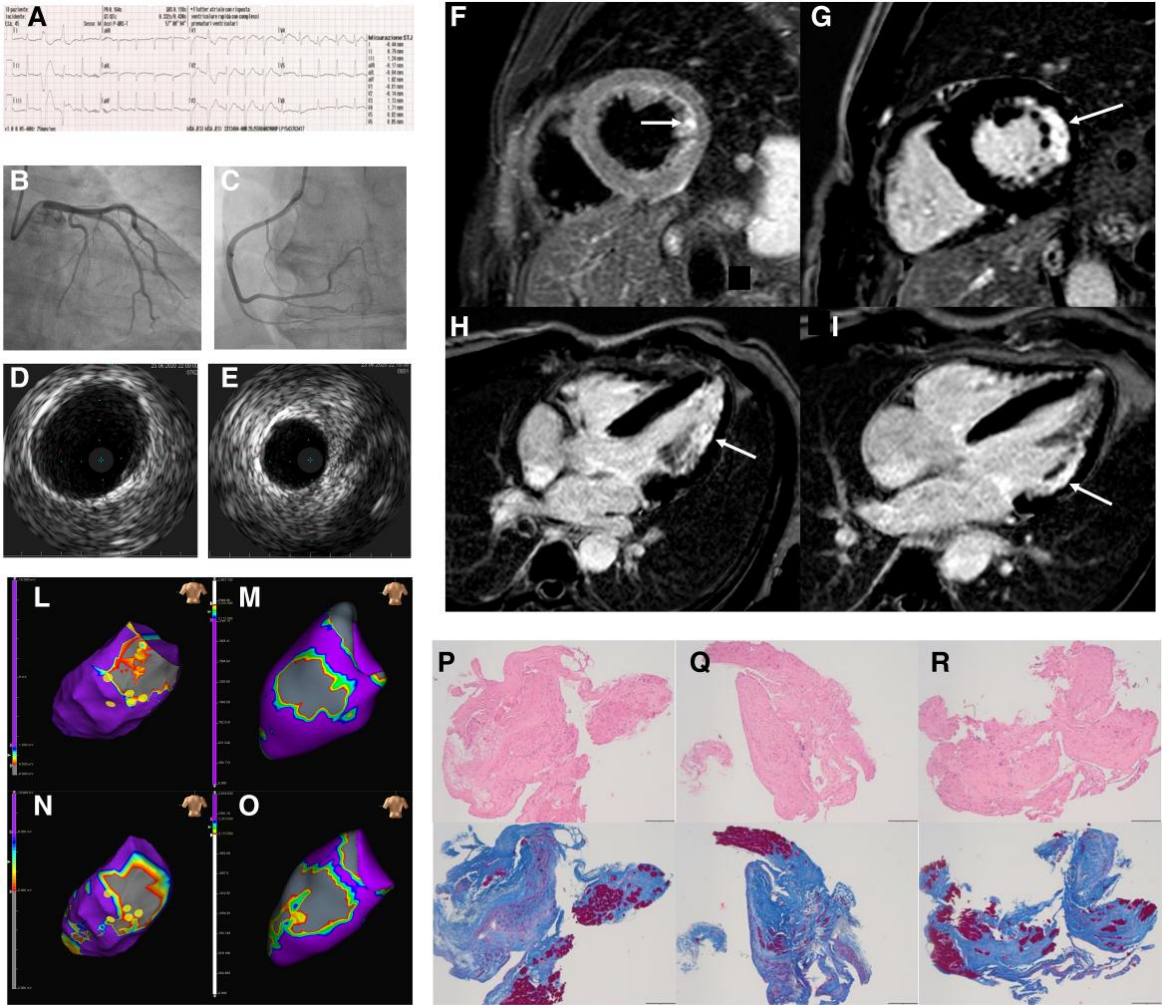


Early cardiovascular magnetic resonance (CMR) showed non-dilated left ventricle (LV) with preserved ejection fraction and hypokinesis of the lateral wall, sub-endocardial oedema in basal and mid-segments of the LV lateral wall on short tau inversion recovery sequences (*Panel F*, arrows) on T2 sequences with corresponding mid-wall-to-sub-endocardium late gadolinium enhancement (LGE) (*Panels G–I*; arrows), puzzling for an ischaemic injury.

After heart-team discussion, we proceed to electroanatomical mapping (EAM)-guided endomyocardial biopsy (EMB) to rule out myocardial inflammation and elucidate CMR findings.

The EAM reconstructions of the LV showed basal inferior-lateral scarring, and 3D pixel signal intensity maps of the LV obtained from LGE CMR sequences with a dedicated software (ADAS VT, Galgo Medical) confirmed scarring of the basal inferior-lateral wall of the LV with a sub-endocardial and mid-myocardial distribution (*Panels L–O*; dots indicates the sites of EMB).

EMB showed massive replacement fibrosis (*Panels P–R*), without evidence of inflammatory infiltrates. Thereafter the patients underwent implantation of a subcutaneous defibrillator.

Furthermore, due to the arrhythmic phenotype, a genetic evaluation was also carried out which revealed an arrhythmogenic cardiomyopathy heterozygous pathogenic variant [NM_024334.3(*TMEM43*):c.1073C > T (p.Ser358Leu)].

In conclusion, even in the presence of advanced imaging tools, a precision-medicine approach is mandatory in order not to miss important pieces in the puzzle, such as genetic mutations.

**Funding:** The authors received no financial support for the research, authorship, and/or publication of this work.

**Data availabilty:** No new data were generated or analysed in support of this research.

## Lead author biography


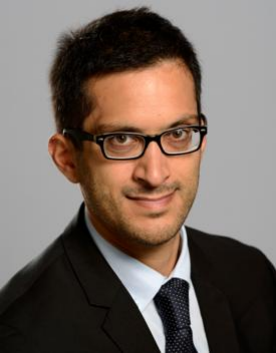


Dr. Giuseppe Ciliberti is a cardiologist skilled in clinical cardiology and integrated cardiac imaging. He currently works as consultant cardiologist at Lancisi Cardiovascular Center, Cardiology and Arrhythmology Unit, Marche University Hospital, Ancona, Italy. He completed his cardiovascular medicine residency at the University of Perugia and Santa Maria della Misericordia Hospital of Perugia in 2016. He completed a clinical and research fellowship at St. George's University of London in 2015, and in 2020 he completed a PhD programme in Biomedical Sciences at Marche Polytechnic University, Ancona, Italy. He was awarded the Masini Award by the Italian National Association of Hospital Cardiologists (ANMCO) in 2017 and he currently is the Chairperson of the ANMCO Young Cardiologists Area. He conducted several studies on myocardial infarction with non-obstructed coronary arteries (MINOCA), which is his main research area of interest, with more than 50 articles published in international peer-reviewed journals. He acts as a reviewer for several cardiology journals. He is a member of the Editorial Board of the ‘International Journal of Cardiology’.

